# *In silico* ADME and Toxicity Prediction of Ceftazidime and Its Impurities

**DOI:** 10.3389/fphar.2019.00434

**Published:** 2019-04-24

**Authors:** Ying Han, Jingpu Zhang, Chang Qin Hu, Xia Zhang, Bufang Ma, Peipei Zhang

**Affiliations:** ^1^Division of Antibiotics, National Institutes for Food and Drug Control, Beijing, China; ^2^Department of Pharmacology, Institute of Medicinal Biotechnology, Chinese Academy of Medical Sciences and Peking Union Medical College, Beijing, China

**Keywords:** ceftazidime, impurity, toxicity, docking, ADME

## Abstract

To improve the quality control of drugs, we predicted the absorption, distribution, metabolism, excretion, and toxicity (ADMET) of ceftazidime (CAZ) and its impurities via *in silico* methods. We used three types of quantitative structure-activity relationship and docking software for precise prediction: Discovery Studio 4.0, OECD QSAR Toolbox 4.1, Toxtree, and the pkCSM approach. The pharmacokinetics and toxicity of ceftazidime and impurity A (Δ-2-CAZ) are similar. The biological properties of impurity B (CAZ *E*-isomer) are different from CAZ. Therefore, we focused on drug stability to analyze impurity B. Impurities D and I have strong lipophilicity, good intestinal absorption, and poor excretion in the body. Impurity D is particularly neurotoxic and genotoxic. It is important to control the content of impurity D. The toxicity of impurity F is low, but the toxicity is enhanced when it becomes the C-3 side chain of CAZ and forms a quaternary amine group. We conclude that the beta-lactam ring of nucleus, the quaternary amine group at the C-3 side chain, and the acetates at the C-7 side chain of CAZ are the main toxic functional groups. Impurities B and D may be the genetic impurity in CAZ and may also have neurotoxicity. This *in silico* approach can predict the toxicity of other cephalosporins and impurities.

## Introduction

Ceftazidime (CAZ) is a third-generation cephalosporin that has been commonly used since the 1990s. It is widely used to treat infections, including sensitive strains and severe infections. In 2015, Ceftazidime-Averbatan was approved by the USFDA for the treatment of complicated urinary tract infections (cUTIs) and combined with metronidazole for the treatment of complicated intra-abdominal infections (cIAIs). CAZ can be administered by intramuscular or intravenous injection with high blood concentration, low protein binding rate (10–17%), renal excretion (80–90%), wide distribution *in vivo*, and blood–brain barrier penetration (25%) (Zasowski et al., 2015; Falcone and Paterson, 2016). CAZ has induced common adverse reactions in the nervous system as well as blood, urinary, and digestive systems. The toxicity of drugs is not only related to the toxicity of active pharmaceutical ingredients (APIs) but also to impurities in drugs (Rayavarapu et al., 2015; Gunther et al., 2017). The drug regulatory agencies of various countries and international organizations require the identification and control of impurities in drugs—especially the control of genetic toxicity impurities (Pikul et al., 2016; Kragelj Lapanja et al., 2017). However, there are few reports on the toxicity of CAZ impurities.

**FIGURE 1:**
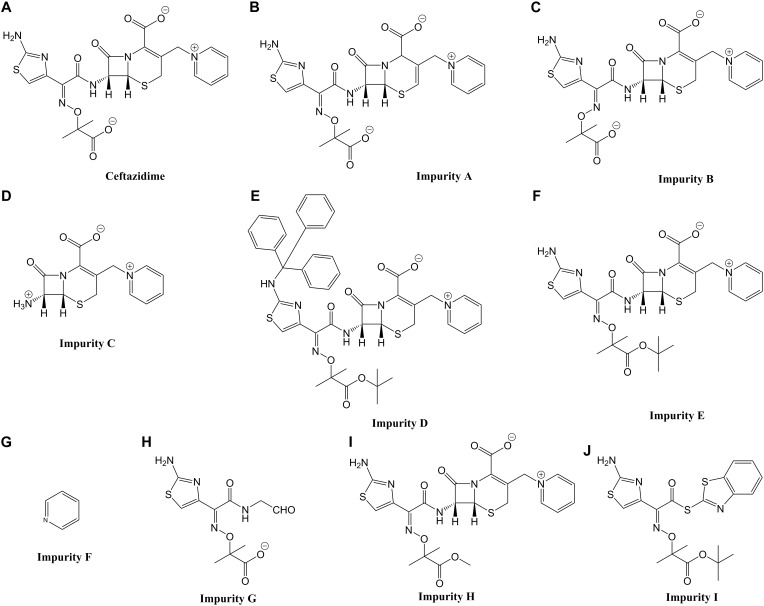
Chemical structures and common names of the 10 compounds investigated in this study. **(A)** Ceftazidime; **(B–J)** impurity A, B, C, D, E, F, G, H, and I.

The toxicity of drug impurities is closely related to their structure. Structure-activity relationships (SARs) have been widely used in Europe and the United States to predict toxicity by computer (Greene et al., 2015; Pavan et al., 2016; Guan et al., 2019). We have recently systematically discussed the structure-toxicity relationship of cephalosporins via the zebrafish embryo toxicity test combined with computational chemistry and molecular docking techniques. This showed that the C-3 and C-7 side chains of cephalosporins are the main toxic functional groups leading to embryo toxicity, neurotoxicity, and cardiotoxicity of cephalosporins and impurities (Zhang et al., 2013; Han et al., 2017, Han B. et al., 2018, Han et al., 2018a; Qian et al., 2018). Therefore, the aim of this study was to systematically summarize the structure-toxicity relationships of cephalosporins and use software to predict the toxicity of ceftazidime impurities listed in Europium pharmacopeia (Figure 1).

## Materials and Methods

### Prediction of ADMET by Computational Analysis

PK properties such as absorption, distribution, metabolism, excretion and toxicity (ADMET) profiling of compounds were determined using the pkCSM ADMET descriptors algorithm protocol^1^ and the Discover Studio 4.0 (DS4.0) software package (Accelrys Software, Inc., San Diego, CA, United States). Two important chemical descriptors correlate well with PK properties, the2D polar surface area (PSA_2D, a primary determinant of fractional absorption) and the lipophilicity levels in the form of atom-based LogP (AlogP98). The absorption of drugs depends on factors including membrane permeability [indicated by colon cancer cell line (Caco-2)], intestinal absorption, skin permeability levels, P-glycoprotein substrate or inhibitor. The distribution of drugs depends on factors that include the blood–brain barrier (logBB), CNS permeability, and the volume of distribution (VDss). Metabolism is predicted based on the CYP models for substrate or inhibition (CYP2D6, CYP3A4, CYP1A2, CYP2C19, CYP2C9, CYP2D6, and CYP3A4). Excretion is predicted based on the total clearance model and renal OCT2 substrate. The toxicity of drugs is predicted based on AMES toxicity, hERG inhibition, hepatotoxicity, and skin sensitization. These parameters were calculated and checked for compliance with their standard ranges.

The prediction of genotoxicity used the OECD QSAR toolbox 4.1 software package (Organization for Economic Co-operation and Development, Paris, France) and Toxtree, Version 2.6.13 (Ideaconsult, Ltd., Sofia, Bulgaria). Both software are open source freely available *in silico* programs that identify the chemical structural alerts (SA).

### Theoretical Studies on Molecular Conformation Analysis in Aqueous Solution

(1) Molecular Mechanical Computation. The possible conformations were generated and minimized by DS 4.0 and the “best” algorithm was applied. The conformer optimization was performed using the CHARMm force field with the implicit solvent model GBMV (generalized born with molecular volume). The dielectric constant of water was set to 80.0. The conformers with lower energies and characteristic features were chosen as the global minima candidates for quantum chemical optimization.

(2) Quantum-mechanical Study. The geometric optimization and thermochemistry calculations were performed by the ORCA 3.0.3 program^2^. The detailed density theory method, basis sets, and parameters were all the same as previously reported (Zhang et al., 2013).

### Molecular Docking

**FIGURE 2:**
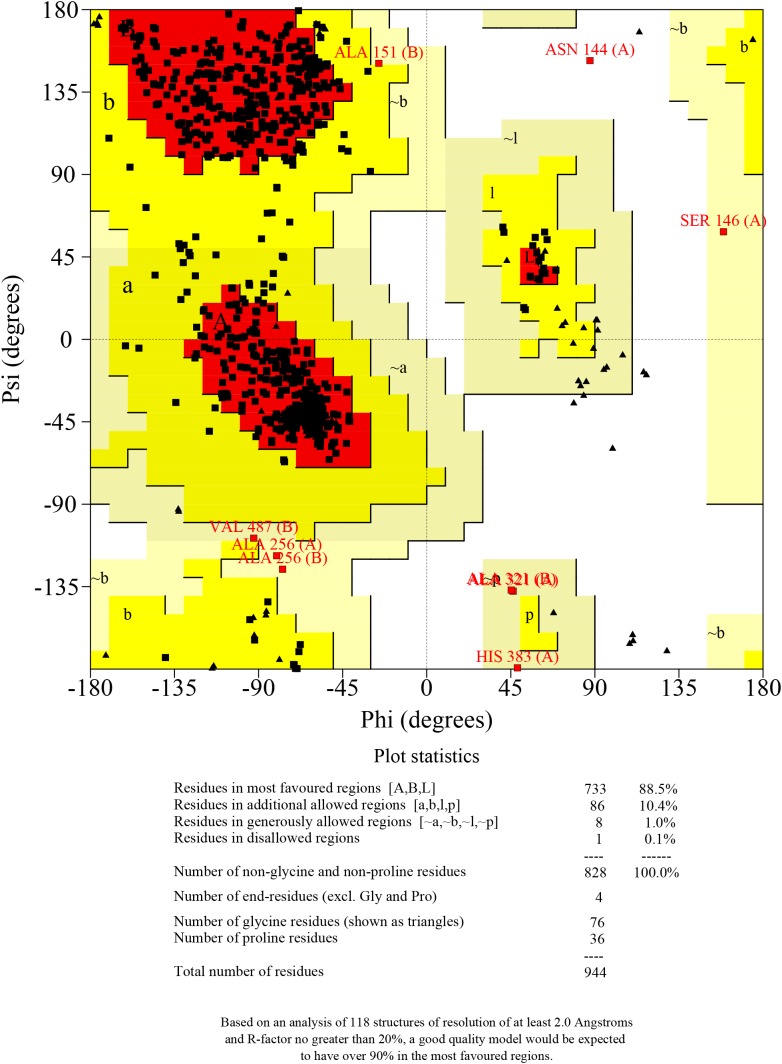
Ramachandran plot for homology model of GRM1A.

The DS4.0 software package was used for docking studies of selected targets and ligands. For protein preparation, a three-dimensional (3D) glutamate receptor metabotropic 1a (GRM1A) was generated through homology modeling server SWISS-MODEL^3^ (Arnold et al., 2006; Biasini et al., 2014). The most suitable template for homology modeling is metabotropic glutamate receptor subtype 1 ligand form I (PDB ID: 1EWT) from rattus norvegicus which shares 83.61% identity with GRM1A. The final 3D structure of GRM1A was evaluated using PROCHECK. The Ramachandran plot obtained showed that 88.5% of residues presented in most favored regions (Figure 2). Before docking, the GRM1A protein was prepared by removing the water molecules and the hydrogen atoms were added to the unoccupied valence of the heavy atoms of the protein. The GRM1A protein was defined as a receptor and the suitable binding site was identified by Define and Edit Binding Site protocol in DS 4.0.

For ligand preparation, the structures of the experimental compounds were downloaded from the PubChem Compound Database^4^. From the receptor–ligand interaction section of DS 4.0, the CDOCKER protocol was selected and used in the docking studies. Docking was performed with a simulated annealing method to minimize the CDOCKER energy and obtain the optimum pose.

## Results

### Analysis of Structural Alerts

OECD QSAR Toolbox 4.1 software was used to predict the structural alerts leading to genotoxicity. Some groups can specifically interact with molecules in the organism. The structural alerts show interactions with genetic material that will induce gene mutation or cause chromosome rearrangement and fragmentation. We predicted the genotoxicity of CAZ and its impurities via the following (Supplementary Table S1): (1) toxicity mechanisms including DNA binding and protein binding, (2) toxicity endpoint including carcinogenicity (genotoxicity), *in vitro* mutagenicity test (Ames test), and *in vivo* mutagenicity (micronucleus test).

For toxicity mechanisms, the DNA-binding groups were imine and positive nitrogen ions in CAZ and impurity A; imine ions in impurities B, C, E, and H; aromatic hydrocarbons in impurity D; and positive nitrogen ions in impurities G and I. Impurity F were predicted to have no structural alerts. The protein-binding groups were acetates and beta-lactam rings in CAZ and impurities A, B, C, D, E, and H; the carbonyl group in impurity G; and acetates in impurity I.

For toxicity endpoints, the carcinogenic groups were aromatic amines as well as hydroxylamines and their derivatives in CAZ and impurities A, B, E, F, G, H, and I. Impurities C and D were predicted to have no structural alerts. The *in vitro* mutagenic groups were aromatic amines, hydroxylamines, and their derivatives in CAZ and impurities A, B, E, G, H, and I; impurities C, D, and F were considered to have no structural alerts. The *in vivo* mutagenic groups were CAZ, aromatic amines, hydroxylamines and their derivatives in impurities A, B, E, G, H and I, and the structure of H-acceptor-path3-H-acceptor as well as the H-acceptor-path3-H-acceptor in impurities C and D. Impurity F were considered to have no structural alerts.

When the potential mutagenicity of impurities was evaluated by Toxtree, impurity F and I were predicted to have two structural alerts, including the heterocyclic, heteroaromatic or a heterocyclic ring with complex substituents, were highlighted (Table 1).

**Table 1:** Structural alerts of compounds using Toxtree analysis.

No	Compound	Fragment	Description
		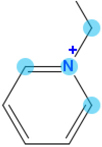	
1	CAZ, Impurity A, B, D, E, H		Quaternary nitrogen
		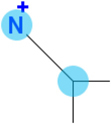	
2	Impurity C		Quaternary nitrogen
		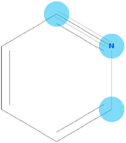	
3	Impurity F		Heterocyclic and heteroaromatic
		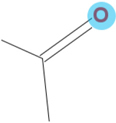	
4	Impurity G		A heterocyclic ring with complex substituents
		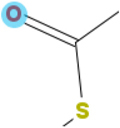	
5	Impurity I		Heterocyclic and a heterocyclic ring with complex substituents


### Rules for Predicting the Toxicity of Cephalosporin Impurities

Based on our studies of the relationship between the structure of cephalosporins and recent embryotoxicity, cardiotoxicity, and neurotoxicity data, we summarize the following general rules for predicting the toxicity of cephalosporin impurities.

Rule 1: The compound toxicity is determined by the structure of its toxic functional group. Compounds with similar structure have similar toxicity. The C-3 side chain and C-7 side chain of cephalosporin are usually different toxic functional groups, and they may have independent or synergistic effects (Zhang et al., 2010, 2013; Han et al., 2018a). By calculating the three-dimensional (3D) structure of cephalosporin, the toxic functional groups of cephalosporin could be predicted.

Rule 2: The toxicity of a compound is not only related to the two-dimensional (2D) structure of toxic functional group but also to its 3D structure. Cephalosporins with two toxic functional groups and one side chain adopted an extended form while the other side chain adopted a more folded form. The extended side chain may appear toxic at first—especially at low concentrations (Zhang et al., 2013; Han et al., 2017).

Rule 3: The degradation of cephalosporins can lead to new toxic functional groups with different toxic effects (Chen et al., 2017; Han et al., 2018b).

Rule 4: Toxic effects of drugs are determined by the characteristics of toxic functional groups and the absorption characteristics of the compounds. The absorption characteristics of drug molecules can be characterized by the space polar area (TPSA). Generally, drugs with smaller TPSA are more easily absorbed (Zhang et al., 2013; Chen et al., 2017).

Rule 5: For the structure characteristics, two common types of the cephalosporins, one is the C-7 side chain with an aminothiazoyl ring, while the other is the C-3 side chain with a methylthiotetrazole (MTT) group. Compared with other structures at the C-7 or C-3 side chains, the acute toxicity based on the structure of aminothixime or MTT is weak (Zhang et al., 2013; Qian et al., 2018).

Rule 6: The spatial structure of toxic functional groups of cephalosporin Δ-isomer does not usually change significantly; its effect on TPSA is small, and the toxic effects of the isomer is similar to the drug (Zhang et al., 2013).

Rule 7: The E-isomer (trans-isomer) of cephalosporin with an aminothiazoyl ring at the C-7 position is more likely to exhibit toxic characteristics of the C-3 side chain because the C-7 side chain is usually folded. Concurrently, the TPSA of the molecule is weakened, and its absorption is enhanced. This can induce acute toxic effects for the E-isomer that are bigger than the original drug (Zhang et al., 2013; Qian et al., 2018).

Rule 8: Cephalosporins with MTT at C-3 position and the C-3 side chains are usually folded. Their toxicity mainly depends on the toxic functional groups of C-7 position (Zhang et al., 2013; Han et al., 2017; Qian et al., 2018).

Rule 9: Cephalosporins with a simple substitution such as methyl, propylene, and chlorine on the C-3 position, the C-3 substituent, and the scaffold (7-ACA) form toxic functional groups together. The toxic functional groups can induce different toxic effects through different mechanisms (Qian et al., 2018).

Rule 10: The neurotoxicity and cardiotoxicity of cephalosporins mainly depend on the toxic functional groups of scaffold and C-3 side chain. When the C-3 side chain is acetoxyl structure, the toxic effect is strong, and the C-7 side chain plays a role in the toxic effect by influencing drug absorption (Chen et al., 2017; Han et al., 2018b).

Rule 11: Cephalosporins may induce neurotoxicity by interacting with glutamate receptor, metabotropic 1a (GRM1A), and/or glutamate decarboxylase 2 (GAD2). Docking with GRM1A or GAD2 proteins may predict the neurotoxicity of cephalosporins (Han et al., 2018b).

Rule 12: Cephalosporins may induce cardiotoxicity by regulating the expression of *nppa*, *adra2c*, and *tnni1c* genes. The protein is encoded by the gene that was used as a receptor to dock with cephalosporins; the results can evaluate the cardiotoxicity of cephalosporins (Han et al., 2018a).

Rule 13: Cephalosporins with MTT at C-3 position may induce embryo toxicity by regulating the expression of *has1* and *cnnm2a* genes. Docking with HAS1 or CNNM2A proteins may predict the embryo toxicity of cephalosporins (Han et al., 2018a).

### 3D Theoretical Structural Calculations

**FIGURE 3:**
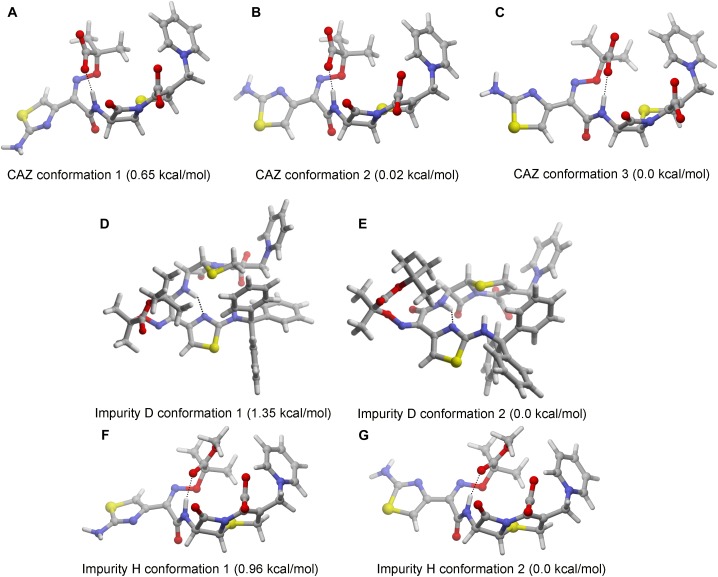
Stable conformations of ceftazidime (CAZ) and impurities in aqueous solution. **(A–C)** CAZ; **(D,E)** impurity D; **(F,G)** impurity H.

The 3D structure of C-3 and C-7 side chains of cephalosporins affect the activity of their toxic functional groups. The 3D structure of impurities D and H cannot be predicted according to the comparison with the structural characteristics of CAZ impurities and the 3D structure of cephalosporins have been calculated. Thus, we performed calculations of the most stable conformations of ceftazidime and impurities D and H in aqueous solution. The results show that CAZ had one intramolecular hydrogen bond between the hydrogen atom of amide nitrogen and the carbonyl of imine group on the C-7 position. Although there may be three stable conformations, both C-3 and C-7 side chains have an extended form (Figure 3A–C). The results indicate that C-3 and C-7 side chains are different toxic functional groups.

Impurity D is a double derivative of amino and carboxyl groups in the aminothixime structure. Although the steric structure has not changed (Figure 3D,E), the disappearance of the aminothixime group forms a new toxic functional group, and the molecular polarity is significantly weakened.

Impurity H is a methyl ester derivative of carboxyl group in the aminothixime structure, and its stable conformation in aqueous solution is similar to the CAZ (Figure 3F,G). The methyl ester group remains essentially unchanged in its steric structure suggesting that its toxic functional groups are similar to CAZ, but the weakness of the molecular polarity makes it easier for absorption.

### Acute Toxicity Prediction

The impurities of CAZ can be classified into three categories according to their structural characteristics: (1) isomers of CAZ including impurity A (Δ-isomer) and impurity B (E-isomer); (2) synthetic initiators/intermediates including impurity C, F, G, and I; and (3) derivatives of CAZ including impurity D, E, and H.

In the zebrafish embryo toxicity test, the half teratogenic rate (TC50) of CAZ was 46.6 mmol/L, the developmental malformations were abdominal abnormalities and blood congestion was at the cardiac inflow region. Furthermore, the bladder of the larvae (5 dpf) was small or absent, most larvae had delayed reaction to tail touch and showed less swimming (Han et al., 2018a,b). The C-3 and C-7 side chains of CAZ in aqueous solution are extensible—that is, the C-3 and C-7 side chains could act as different toxic functional groups. Thus, the acute toxicity based on the C-7 side chain is weak, suggesting that the toxic effect of CAZ mainly depends on the toxic functional groups at the C-3 side chain. Thus, the toxicity of CAZ impurities can be predicted according to the toxicity prediction rules of cephalosporins and the zebrafish toxicity test results of CAZ.

The structure of impurities F and G are similar to the C-3 and C-7 side chains of CAZ, respectively. They can also be regarded as synthetic precursors of CAZ. Impurity G belongs to the toxic functional group based on the structure of an aminothiazoyl ring at the C-7 position. Thus, the toxicity of impurity G is weak according to Rule 6. The toxicity of impurity F (pyridine) can be consulted in the toxicity database of compounds without using the zebrafish toxicity test.

Impurity I is a by-product of synthesis. Although it contains a complete C-7 side chain of CAZ, the binding structure of benzothiazole not only changes the molecular polarity of CAZ but also forms new toxic functional groups. Thus, the current rules for predicting the toxicity of cephalosporin cannot predict its toxicity.

Impurity A is the Δ-isomer of CAZ. Rule 5 states that the toxicity of the Δ-isomer and drug are similar so the toxicity of impurity A is similar to CAZ.

Impurity B is the E-isomer of CAZ. According to Rule 7, the toxic effect of CAZ E-isomer is mainly based on C-3 side chain structure, and it is more easily absorbed. Therefore, the toxicity of impurity B may be similar to CAZ, but the toxic effect is enhanced. The TD50 of cefotaxime is 30 mmol/L, its E-isomer is 6 mmol/L, the TD50 of ceftriaxone is 17 mmol/L, and the E-isomer of ceftriaxone is 3.4 mmol/L (Zhang et al., 2013). This suggests that the toxicity effect of E-isomer is no more than 10 times that of CAZ.

Impurity C is an intermediate formed by the combination of the 7-ACA and C-3 side chains of CAZ. Rule 9 states that impurity C can induce neurotoxicity and cardiotoxicity.

Impurity H is the methyl ester of CAZ. The 3D structure analysis reveals that its toxic functional group is similar to CAZ, but its molecular polarity is weak. This makes it easier absorbed. Therefore, the toxicity of impurity H may be similar to CAZ, but the toxic effect is stronger. The TC50 of impurity H is 1.9 mmol/L, which is about 20 times lower than that of CAZ. The abnormal phenotype of the embryo at low concentrations is similar to CAZ. At high concentrations, the embryo body is shorter and curved, the body is dark, the notochord is abnormal, and the yolk sac extension shorter and thicker (Sun et al., 2014). This verified the prediction results.

Impurity E is a tertiary methyl butyl ester derivative of CAZ. Compared with impurity H, tertiary methyl butyl ester has stronger hydrophobicity and weaker molecular polarity, but its toxic functional group may be similar with impurity H. The toxic effect of impurity E may be stronger than impurity H.

The 3D structure of impurity D reveals that the derivation of amino group at the C-7 position forms a new toxic functional group; however, the toxic functional group at the C-3 position is similar to CAZ. In addition, the polarity of impurity D significantly decreases due to the double derivatization. Thus, impurity D may be easily absorbed by the body. The toxicity effects of impurity D could not be predicted accurately according to the current toxicity prediction rules.

In conclusion, the zebrafish toxicity prediction results indicated that the toxicity types of CAZ impurities were similar to that of ceftazidime, but some impurities were more toxic than CAZ because they were more easily absorbed. However, the toxic effects of impurity D could not be predicted due to the formation of new toxic functional groups. In addition, the neurotoxicity of impurity C should be further evaluated through docking experiments.

### Neurotoxicity Prediction

**FIGURE 4:**
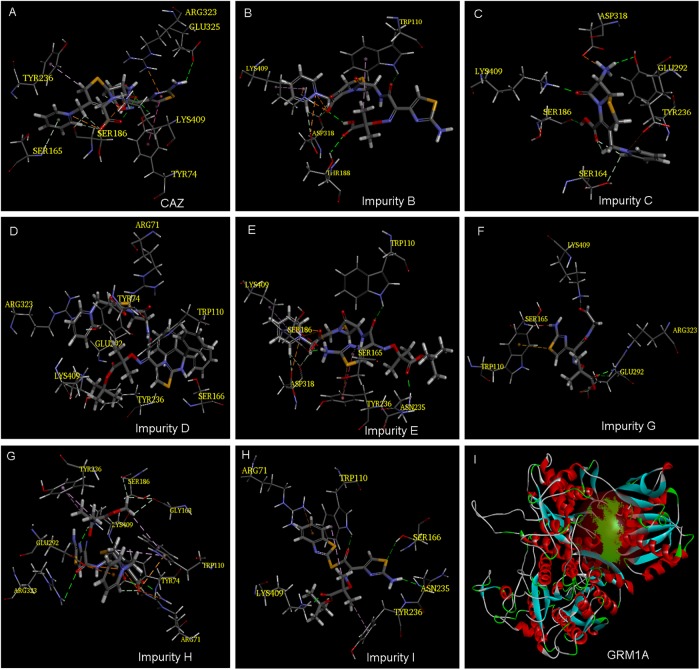
Model of ligand docking. 3D structure showing **(A–H)** CAZ, impurity B, impurity C, impurity D, impurity E, impurity G, impurity H, and impurity I interactions with the residues of GRM1A; best docking poses of compound inside the GRM1A binding sites showing the different interactions. Arrow: hydrogen bond (HB) formed with the side chain of the residue. **(I)** The active site of GRM1A.

Our previous study showed that the cephalosporins with an aminothiazoyl ring at the C-7 position may have neurotoxic effects in zebrafish by interacting with glutamate receptor metabotropic 1a (GRM1A) protein (Figure 4I) (Han et al., 2018b). The docking results tabulated between the GRM1A protein and the compounds are shown in Figure 4 and Table 2. The results showed that the carboxyl and carbonyl groups on the scaffold of CAZ formed hydrogen bonds with residues LYS409 and TYR74, and the thiazole group at the C-7 position of CAZ formed pi interactions with the residue ARG323 (Figure 4A). The carboxyl groups on the scaffold of impurity B formed hydrogen bonds with the LYS409 residue. The positive nitrogen ion at the C-3 position formed hydrogen bonds with the ASP318 residue, and the pyridine ring formed pi bonds with the LYS409 residue. The carbonyl group on the C-7 position formed hydrogen bond with the THR188 residue (Figure 4B). Hydrogen and carbonyl groups on the carboxyl group of the scaffold of impurity C formed hydrogen bond with SER186 and LYS409, respectively. The amino group at the C-7 position formed hydrogen bond with residues TYR236 and ASP318 (Figure 4C). The carboxyl and carbonyl groups of impurity D scaffold formed hydrogen bond with ARG71 residue, and the pyridine ring on the C-3 position formed pi interactions with ARG323. The oxygen atom of the tertiary butyl ester on the C-7 side chain formed hydrogen bond with the LYS409 residue (Figure 4D). The carboxyl group on the scaffold of impurity E formed hydrogen bonds with the LYS409 residue. The positive nitrogen ion on the C-3 position formed hydrogen bonds with the ASP318 residue, and the pyridine ring formed pi interactions with the LYS409 residue. The carbonyl and amino groups on the C-7 position formed hydrogen bonds with ASN235 and SER186, respectively (Figure 4E). The carbonyl group of impurity G formed hydrogen bond with the LYS409 residue. The carboxyl group formed hydrogen bonds with ARG323 and GLU292 residues (Figure 4F). Carboxyl and carbonyl groups on the scaffold of impurity H formed hydrogen bond with ARG71 residue, and the positive nitrogen ion on the C-3 position formed a hydrogen bond with residue GLU292, and carbonyl and amino on the C-7 position formed hydrogen bond with LYS409 residue. The thiazole ring formed pi interactions with the ARG323 residue (Figure 4G). Carbonyl and tert-butyl acetate groups of impurity I formed hydrogen bonds with TRP110 and LYS409 residues, respectively (Figure 4H).

**Table 2:** Dock results.

Compound	-CDOCKER energy	-CDOCKER interaction energy
CAZ	19.041	56.257
Impurity B	20.465	57.077
Impurity C	6.012	43.413
Impurity D	20.558	67.445
Impurity E	32.974	62.828
Impurity G	36.279	39.662
Impurity H	24.559	64.189
Impurity I	27.386	39.123


The analysis of docking experiments also showed that impurity G presented with the highest -CDOCKER energy (36.279) inside the GRMIA binding cavity, while the lowest energy was reported for impurity C (6.012). This suggests that impurity G may be the highest neurotoxicity compound (Table 2).

### Prediction of ADMET Properties

The ADMET properties of CAZ and its impurities are presented in Table 3. The PSA_2D is closely related to the absorption properties of compounds. The PSA_2D of CAZ as well as impurities A, B, D, E, G, and H were all greater than 140, suggesting that these compounds had strong polarity and were not easily absorbed by the body. The PSA_2D of impurities C and F were all less than 100 indicating that two compounds had good oral absorption or membrane permeability (Qidwai, 2016). CAZ and impurities A, B, C, E, F, G, and H were predicted as having ideal lipophilicity (AlogP98 ≤ 5), impurity D and I were shown as poor lipophilicity (AlogP98 > 5) (Fonteh et al., 2015). The result suggested that impurity D and I have poor absorption and permeation.

**Table 3:** Predicted ADMET properties of compounds.

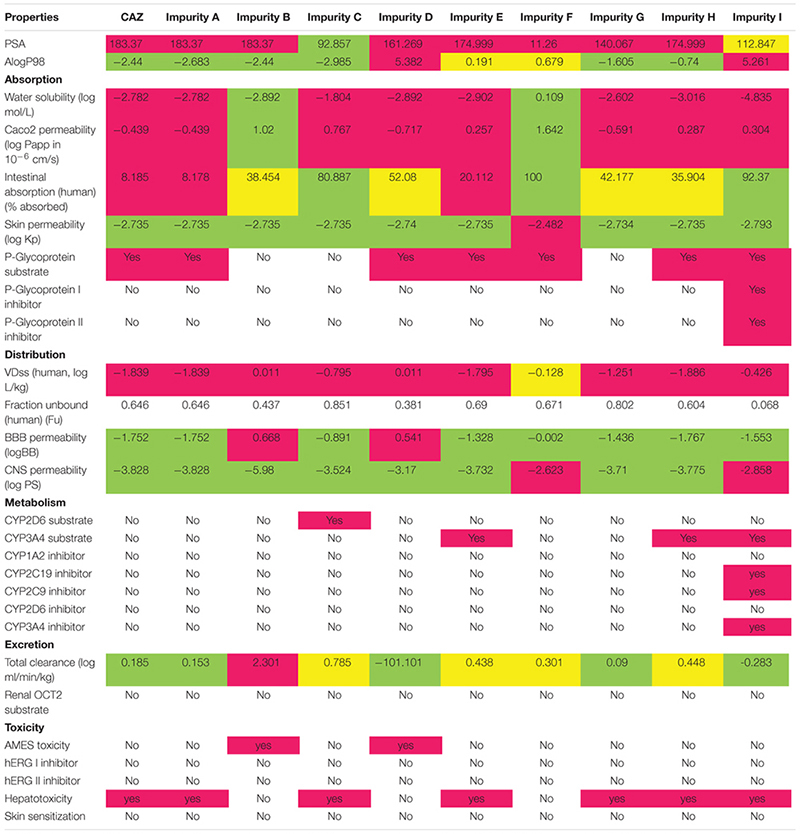

*ADMET, absorption, distribution, metabolism, excretion, and toxicity; Papp, apparent permeability coefficient; AMES, assay of the ability of a chemical compound to induce mutations in DNA; Kp, skin permeability constant; Fu, fraction unbound; BBB, blood–brain barrier; BB, blood–brain; CNS, central nervous system; PS, permeability-surface area; T. pyriformis, Tetrahymena pyriformis; LD, lethal dose; LOAEL, lowest-observed-adverse-effect level.*

Caco-2 permeability, intestinal absorption (human), skin permeability, and P-glycoprotein substrate or inhibitor were used to predict the absorption level of the compounds. When the Papp coefficient is >8 × 10^-6^, the predicted value is >0.90; thus, the compound has high Caco-2 permeability and is easy to absorb. Impurity B and F were predicted to have high Caco-2 permeability. With regards to intestinal absorption (human), absorbance of less than 30% is considered to be poorly absorbed. CAZ and impurity A and E were predicted to have a poor absorption. With regards to skin permeability, the log Kp > -2.5, the compound is considered to be relatively low skin permeability. Impurity F was considered to have low skin permeability. P-glycoprotein is a member of the ATP-binding transmembrane glycoprotein family [ATP-binding cassette (ABC)], which can excrete drugs or other exogenous chemicals from cells. The results suggested that CAZ and impurities A, D, E, F, H, and I are all substrates of P-glycoprotein—they may be actively exuded from cells by P-glycoprotein. Impurity I was predicted to be a P-glycoprotein inhibitor.

The distribution volume (VDss), Fraction unbound (human), CNS permeability and blood–brain barrier membrane permeability (logBB) were used to characterize the distribution of compounds. The distribution volume is a parameter to characterize the distribution of drugs in various tissues *in vivo*. When the VDss is lower than 0.71 L kg^-1^ (log VDss < -0.15), the distribution volume is considered to be relatively low. When VDss is higher than 2.81 L kg^-1^ (log VDss > 0.45), the distribution volume is considered to be relatively high. The results showed that the distribution volume of CAZ and other impurities were low; however, the VDss of impurity F was -0.128 > -0.15. For blood–brain barrier membrane permeability, logBB > 0.3, the compounds were thought to cross the blood–brain barrier easily. A logBB < -1 suggested that the compounds did not easily cross the blood–brain barrier. CAZ and impurities A, E, G, H, and I were predicted to difficultly cross the blood–brain barrier, but impurities B and D easily crossed. For CNS permeability, CAZ and impurities A, B, C, D, E, G, H, and I were predicted to be unable to penetrate the CNS (logPS is < -3), while impurity F and I may penetrate the CNS.

Cytochrome P450s is an important enzyme system for drug metabolism in liver. The two main subtypes of cytochrome P450 are CYP2D6 and CYP3A4. The results showed that CAZ and impurities A, B, D, F, and G were not substrates for the two subtypes; impurity C was a substrate for CYP2D6, and impurities E, H, and I were substrates for CYP3A4. Impurity I was predicted to be CYP2C19 inhibitor, CYP2C9 inhibitor, and CYP3A4 inhibitor. This suggested that impurities C, E, H, and I may be metabolized in the liver.

Drug elimination is related to the molecular weight and hydrophilicity of compounds. The prediction results show that the total clearance of impurity B is the highest followed by impurities C, H, E, F, CAZ, A, G, I, and D.

The results also suggest that impurity B and D may be toxic in AMES test; CAZ and impurities A, C, E, G, H, and I may be hepatotoxic; but CAZ and its impurities may not inhibit the hERG channel and may not have cardiotoxicity or skin sensitization.

Thus, the predicted results indicate that the ADMET characteristics of most CAZ impurities are similar with those of CAZ. However, impurity D was not easily metabolized and cleared because of its strong lipophilicity. It can easily cross the blood–brain barrier and may have AMSE toxicity. This should be a focus of attention. In addition, impurity I has strong lipophilicity, and may cross the blood–brain barrier and leading to neurotoxicity.

#### Color Coding of Test Chemical Properties

The predicted properties are color coded to facilitate identification among the different chemicals. The color codes include: pink for highly positive, yes; yellow for weak positive; green for negative, no.

## Discussion

The structure of a compound determines its physical and chemical properties as well as the ADMET. Here, the ADMET parameters of CAZ and its nine impurities were studied by two predictive software, and the structural alerts of their possible genotoxicity were predicted, based on three toxicology database. The relationship between the structure and toxicity of CAZ and its impurities was preliminarily evaluated.

The impurities were classified into two categories: β-lactam structure (I) and non-β-lactam structure (II). The impurities were further classified into two sub-categories: process impurities (A) and degradation impurities (B). Class I A are process impurities with a β-lactam structure, including impurities C, D, E, and H. Impurity C is a synthetic intermediate. It is not a substrate of P-glycoprotein and is not easily transported; it may be metabolized in the liver and has hepatotoxicity. Impurity D is a by product with strong lipophilicity; it is the substrate of P-glycoprotein and is easily transported. It can easily cross the blood–brain barrier leading to neurotoxicity, and may have genotoxicity. Impurities E and H are by products and may be metabolized in the liver and have hepatotoxicity.

Our previous zebrafish embryo toxicity tests showed that the mortality and teratogenicity rates of impurity H were significantly higher than those of CAZ (Sun et al., 2014). Class I B are degradable impurities with a β-lactam structure, including impurities A and D. CAZ and impurity A (Δ-2-CAZ) have similar ADMET parameters and warning structures of genotoxicity. They are the substrates of P-glycoprotein and are easily transported in the body and may have hepatotoxicity. They are isomers, but their biological activities may not be very different. Impurity B is a CAZ E-isomer with good Caco 2 permeability that is easily absorbed. It can cross the blood–brain barrier and may have neurotoxicity. It may have genetic toxicity.

Class II A are the process impurities with a non-β-lactam structure, including impurities F and I. Impurity F is the precursor of the C-3 side chain of CAZ and is easily absorbed and transported in the body. Impurity I is an active ester of the C-7 side-chain with strong lipophilicity. It may be absorbed by the intestinal tract and metabolized by liver drug enzymes and may have hepatotoxicity and genotoxicity. Class II B is a degradation impurity with a non-β-lactam structure, including impurity G. It is the C-7 side chain of CAZ, may be easily absorbed but not transported and have hepatotoxicity.

**FIGURE 5:**
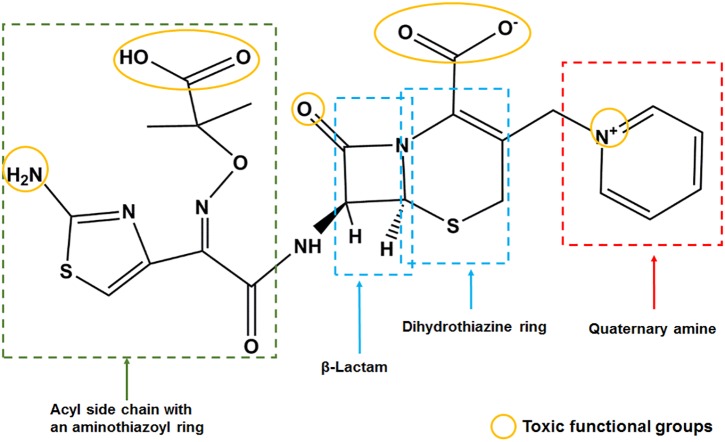
Schematic representation of toxic functional groups of ceftazidime (CAZ) and impurities.

The molecular docking results suggest that the scaffold of CAZ and its impurities, the quaternary amine group on the C-3 position, and the carboxyl and amino groups on the C-7 position may form hydrogen bonds with GRM1A protein residues and lead to neurotoxic effects. Prediction of the structural alerts of genotoxicity also showed that the genotoxicity of CAZ and its impurities were mainly related to aromatic amine groups. The positive nitrogen ion (quaternary amine structure) can easily bind to DNA, and its β-lactam ring on the scaffold and acetic acid (salt) group on the C-7 position can easily bind to proteins inducing toxicity (Figure 5).

## Conclusion

Pyridine itself may be not toxic, but the N atom on the pyridine ring links with methyl to form a quaternary amine group that becomes the toxic C-3 substituent of CAZ. In addition, the E-isomer is important in CAZ quality. The formation of CAZ E-isomer is related to light and other factors. Therefore, the stability of CAZ E-isomer is critical. Impurity D may be especially neurotoxic and genotoxic. Impurity I may have hepatotoxicity. They are the key contaminant to control. In summary, the main toxic functional groups of CAZ and its impurities are the β-lactam ring of the scaffold, the quaternary amine group of the C-3 side chain, and the acetic acid (salt) on the C-7 side chain. This information provides a theoretical and experimental basis for predicting the toxicity of cephalosporins and their impurities, and for the quality control of these impurities.

## Author Contributions

JZ and CH conceived, designed, and supervised the study. YH performed the ADMET prediction and docking experiments and analyzed the data. YH and CH wrote the manuscript. XZ performed the molecular conformation analysis. BM and PZ performed the ADMET prediction.

## Conflict of Interest Statement

The authors declare that the research was conducted in the absence of any commercial or financial relationships that could be construed as a potential conflict of interest.

## Footnotes

1 http://biosig.unimelb.edu.au/pkcsm/prediction

2 https://orcaforum.cec.mpg.de/

3 https://swissmodel.expasy.org/

4 https://www.ncbi.nlm.nih.gov/pccompound/
