# Supplementary material for: In silico ADME and Toxicity Prediction of Ceftazidime and Its Impurities
Source: Front Pharmacol. 2019 Apr 24;10:434. doi: 10.3389/fphar.2019.00434 (PMC6491819; doi:10.3389/fphar.2019.00434)
Supplement: Supplementary file 1 [file Table_1.DOCX]

**Table S1 Results of OECD QSAR toolbox searched for structural alerts**.

|  | **General Mechanistic** | | **Endpoint Specific** | | |
| --- | --- | --- | --- | --- | --- |
|  | **DNA binding by OECD** | **Protein binding by OECD** | **Carcinogenicity (genotox and nongenotox) alerts by ISS** | **in vitro mutagenicity (Ames test) alerts by ISS** | **in vivo mutagenicity (Micronucleus) alerts by ISS** |
| **CAZ** | SN1 >> Iminium Ion Formation >> Aliphatic tertiary amines;  SN1 >> Nitrenium Ion formation >> Primary (unsaturated) heterocyclic amine | Acylation >> Direct Acylation Involving a Leaving group >> Acetates;  Acylation >> Ring Opening Acylation >> beta-Lactams | Primary aromatic amine,hydroxyl amine and its derived esters (Genotox) | Primary aromatic amine,hydroxyl amine and its derived esters | Primary aromatic amine, hydroxyl amine and its derived esters;  H-acceptor-path3-H-acceptor |
| **Impurity A** | SN1 >> Iminium Ion Formation >> Aliphatic tertiary amines;  SN1 >> Nitrenium Ion formation >> Primary (unsaturated) heterocyclic amine | Acylation >> Direct Acylation Involving a Leaving group >> Acetates;  Acylation >> Ring Opening Acylation >> beta-Lactams | Primary aromatic amine,hydroxyl amine and its derived esters (Genotox) | Primary aromatic amine,hydroxyl amine and its derived esters | Primary aromatic amine, hydroxyl amine and its derived esters; H-acceptor-path3-H-acceptor |
| **Impurity B** | SN1 >> Iminium Ion Formation >> Aliphatic tertiary amines | Acylation >> Direct Acylation Involving a Leaving group >> Acetates;  Acylation >> Ring Opening Acylation >> beta-Lactams | Primary aromatic amine,hydroxyl amine and its derived esters (Genotox) | Primary aromatic amine,hydroxyl amine and its derived esters | H-acceptor-path3-H-acceptor;  Primary aromatic amine, hydroxyl amine and its derived esters |
| **Impurity C** | SN1 >> Iminium Ion Formation >> Aliphatic tertiary amines | Acylation >> Direct Acylation Involving a Leaving group >> Acetates;  Acylation >> Ring Opening Acylation >> beta-Lactams | No alert found | No alert found | H-acceptor-path3-H-acceptor |
| **Impurity D** | Michael addition >> P450 Mediated Activation to Quinones and Quinone-type Chemicals >> Arenes | Acylation >> Direct Acylation Involving a Leaving group >> Acetates;  Acylation >> Ring Opening Acylation >> beta-Lactams | No alert found | No alert found | H-acceptor-path3-H-acceptor |
| **Impurity E** | SN1 >> Iminium Ion Formation >> Aliphatic tertiary amines | Acylation >> Direct Acylation Involving a Leaving group >> Acetates;  Acylation >> Ring Opening Acylation >> beta-Lactams | Primary aromatic amine,hydroxyl amine and its derived esters (Genotox) | Primary aromatic amine,hydroxyl amine and its derived esters | H-acceptor-path3-H-acceptor  Primary aromatic amine, hydroxyl amine and its derived esters |
| **Impurity F** | No alert found | No alert found | Primary aromatic amine,hydroxyl amine and its derived esters (Genotox)  Simple aldehyde (Genotox) | No alert found | No alert found |
| **Impurity G** | SN1 >> Nitrenium Ion formation >> Primary (unsaturated) heterocyclic amine | Schiff Base Formers >> Direct Acting Schiff Base Formers >> Mono-carbonyls | Primary aromatic amine,hydroxyl amine and its derived esters (Genotox) | Primary aromatic amine,hydroxyl amine and its derived esters  Simple aldehyde | H-acceptor-path3-H-acceptor;  Primary aromatic amine, hydroxyl amine and its derived esters; Simple aldehyde |
| **Impurity H** | SN1 >> Iminium Ion Formation >> Aliphatic tertiary amines | Acylation >> Direct Acylation Involving a Leaving group >> Acetates;  Acylation >> Ring Opening Acylation >> beta-Lactams | Primary aromatic amine,hydroxyl amine and its derived esters (Genotox) | Primary aromatic amine,hydroxyl amine and its derived esters | H-acceptor-path3-H-acceptor  Primary aromatic amine, hydroxyl amine and its derived esters |
| **Impurity I** | SN1 >> Nitrenium Ion formation >> Primary (unsaturated) heterocyclic amine | Acylation >> Direct Acylation Involving a Leaving group >> Acetates | Primary aromatic amine,hydroxyl amine and its derived esters (Genotox) | Primary aromatic amine,hydroxyl amine and its derived esters | H-acceptor-path3-H-acceptor  Primary aromatic amine, hydroxyl amine and its derived esters |
